# Predicting Postoperative Myopic Shift After Paediatric Intraocular Lens Implantation: A Scoping Review of Associated Factors

**DOI:** 10.3390/medicina62010106

**Published:** 2026-01-03

**Authors:** Ivana Mravičić, Emma Grace Orešković, Maja Bohač, Nataša Drača

**Affiliations:** 1 Svjetlost Eye Clinic, 10000 Zagreb, Croatia; ivana.mravicic@svjetlost.hr (I.M.); maja.bohac@svjetlost.hr (M.B.); natasa.draca@svjetlost.hr (N.D.); 2 Queen Square Institute of Neurology, University College London, London WC1N 3BG, UK

**Keywords:** paediatric cataract, intraocular lens implantation, myopic shift, pseudophakia, axial length, refractive prediction, biometry

## Abstract

*Background and Objectives:* Predicting postoperative refractive development after paediatric intraocular lens (IOL) implantation remains challenging due to continued ocular growth and interindividual variability. This scoping review maps current evidence on demographic, biometric, and surgical factors influencing postoperative myopic shift in children undergoing cataract surgery with IOL implantation. *Methods and Materials:* A systematic literature search was conducted in PubMed and Scopus from the last ten years through October 2025. Eligible studies included children (≤18 years) with congenital or developmental cataract undergoing primary or secondary IOL implantation that reported postoperative refractive change and its predictors. Titles, abstracts, and full texts were screened according to PRISMA-ScR guidelines. Data were charted on study design, age at surgery, follow-up duration, refractive and biometric outcomes, and associated predictors. *Results:* Twelve studies met the inclusion criteria. Younger age at surgery, shorter preoperative axial length, and unilateral cataract consistently predicted greater postoperative myopic shift. Reported myopic change ranged from approximately −1.8 D after 2 years to −11.6 D after 15 years of follow-up, correlating with the rate of axial elongation. Optical biometry and modern formulas (e.g., Holladay 1) showed lower absolute prediction error than manual A-scan or SRK-II calculations. Postoperative complications, especially glaucoma and visual axis opacification, were associated with greater refractive change. *Conclusions:* Postoperative myopic shift is a predictable, age-dependent feature of paediatric pseudophakia driven primarily by ocular growth dynamics. Standardised biometry, age-stratified refractive targeting, and integration of longitudinal growth models into IOL calculation algorithms may improve refractive predictability and visual outcomes in children.

## 1. Introduction

Implantation of an IOL in the developing eye introduces a unique refractive challenge. During childhood, ocular growth is characterised by progressive axial elongation and corneal flattening, which alter the effective power of a fixed IOL and often lead to a progressive postoperative myopic shift [1,2]. This phenomenon is particularly pronounced in infants and young children, where the rate of ocular growth is rapid and unpredictable [1,2,3]. Despite advances in biometric measurement and IOL power calculation formulas, accurately predicting the magnitude of postoperative refractive change remains a significant challenge in paediatric cataract surgery [2,4,5].

To minimize postoperative myopia, surgeons typically aim for a residual hyperopic refraction at the time of implantation [6]. The intended under-correction is age-adjusted and based on empirical estimates of future eye growth [7,8]. However, the degree of myopic shift varies widely among children of similar age and baseline biometry. Factors such as axial length, keratometry, age at surgery, cataract morphology, and the timing of IOL implantation (primary versus secondary) have been variably implicated, but findings across the literature remain inconsistent [1,2,3,4,7,9]. These variations stem in part from differences in surgical technique, follow-up duration, IOL power calculation formulas, and target refractions, as well as from heterogeneous study designs and small sample sizes [2,6,7,10].

Accurate prediction of postoperative refractive development is clinically important for several reasons. Excessive myopic shift can result in anisometropia, amblyopia, and reduced visual acuity, while insufficient under-correction may lead to early hyperopic defocus, where the focal point of the optical system lies behind the retina, causing blurred retinal images and hindering visual rehabilitation [11]. Furthermore, understanding the interaction between biometric parameters and postoperative refractive outcomes can inform the selection of age- and biometry-specific IOL constants and support the refinement of formula adjustments in paediatric eyes [9,10,11].

Previous reviews have summarised refractive outcomes after paediatric IOL implantation but have not comprehensively mapped the parameters most consistently associated with myopic shift [12]. Given the growing number of long-term longitudinal cohorts and improved biometric measurement techniques, a structured synthesis is now warranted.

This scoping review aims to identify studies evaluating predictive factors for postoperative myopic shift in children undergoing primary or secondary IOL implantation. Specifically, this review identifies (1) demographic and biometric variables correlated with the extent of myopic shift, (2) the influence of surgical timing, IOL type, and calculation formula, and (3) the methodological heterogeneity among studies reporting these outcomes. By consolidating these findings, this review provides a framework for optimising IOL power selection and guiding future predictive modeling in paediatric pseudophakia.

## 2. Materials and Methods

### 2.1. Study Design

This scoping review was conducted according to the methodological framework following the Preferred Reporting Items for Systematic Reviews and Meta-Analyses extension for Scoping Reviews (PRISMA-ScR). The aim was to identify and map all available evidence describing predictive factors of postoperative myopic shift following IOL implantation in children with congenital, developmental, or paediatric cataract.

Generative artificial intelligence tools were used in a limited manner to assist with language editing and improvement of clarity. These tools were not used for study selection, data extraction, data analysis, interpretation of results, or generation of scientific content. All methodological decisions, analyses, and conclusions were made by the authors.

### 2.2. Search Strategy

A comprehensive electronic search of PubMed and Scopus was conducted to identify studies published over the last decade evaluating refractive and biometric outcomes after paediatric IOL implantation. Search terms were designed to capture four conceptual domains: (1) paediatric populations; (2) pseudophakia or IOL implantation; (3) refractive or axial outcomes; (4) determinants or predictors of refractive change. Boolean operators and field tags were adapted for each database.

**Figure 1:**
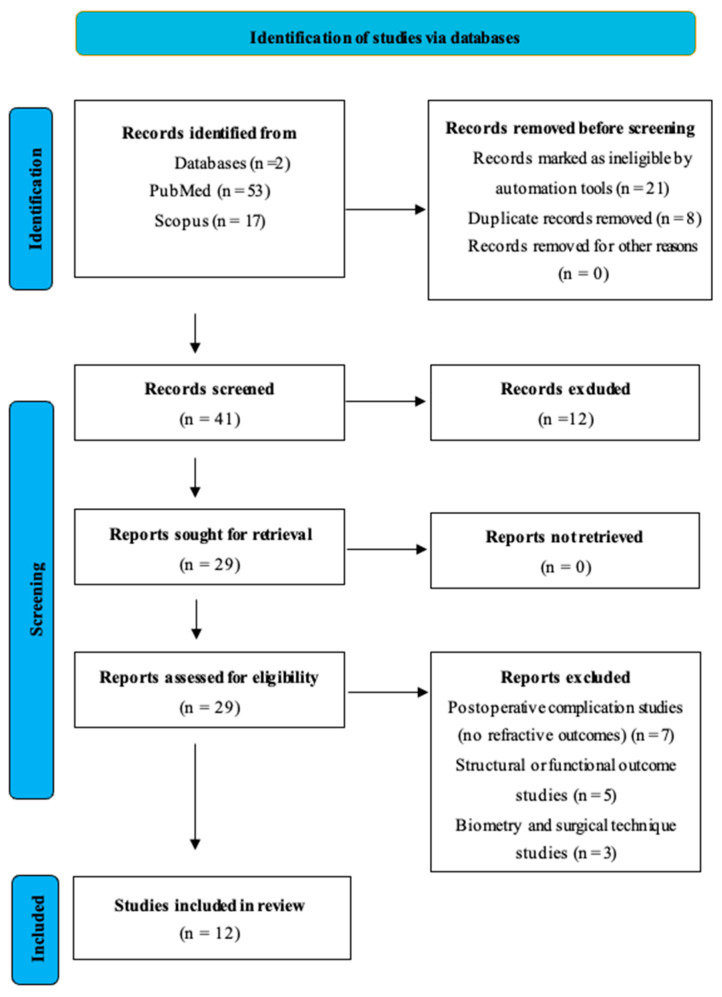
Flow diagram of the study selection process. A total of 70 records were identified (53 from PubMed and 17 from Scopus). After removal of 29 ineligible or duplicate records, 41 were screened, 29 were assessed for eligibility, and 12 studies met the inclusion criteria and were included in the final synthesis.

The detailed PubMed and Scopus search strategies, including applied filters and query strings, are provided on the Open Science Framework (OSF; DOI: [https://doi.org/10.17605/OSF.IO/8PTGJ] (accessed on 3 December 2025)). The stepwise manner of the search strategy employed is outlined in Figure 1. Briefly, the PubMed search initially retrieved 53 records, which were reduced to 32 after the following filters were applied: last 10 years, English language, Humans, Child (birth–18 years), exclude preprints, and MEDLINE. The corresponding Scopus search yielded 17 records after limiting to studies published in English since 2015.

All retrieved references were exported to EndNote 21.5 (Clarivate Analytics, Philadelphia, PA, USA), which was used as an automated reference management and analysis tool to identify and remove duplicates. The deduplicated dataset was then screened for eligibility.

### 2.3. Eligibility Criteria

Studies were eligible if they:

1. Included children ≤ 18 years undergoing primary or secondary IOL implantation for congenital, developmental, or paediatric cataract.
2. Reported quantitative postoperative refractive outcomes (spherical equivalent refraction or myopic shift, in diopters) and/or axial length change (in millimeters).
3. Examined determinants, predictors, or risk factors associated with postoperative refractive change.
4. Were original, peer-reviewed articles published in English.

Exclusion criteria were:

- Studies focusing solely on postoperative complications (e.g., glaucoma, retinal detachment) without refractive data.
- Traumatic, syndromic, or PFV-associated cataracts.
- Reviews, case reports, technical notes, or animal studies.
- Lack of quantitative refractive outcomes or follow-up duration.

### 2.4. Study Selection

Two reviewers independently screened all titles and abstracts for relevance (EO, IM), followed by full-text assessment against eligibility criteria (ND, MB). Disagreements were resolved by discussion and consensus. The selection process was documented in a PRISMA flow diagram (refer to Figure 1).

### 2.5. Data Charting

Data from each eligible study were extracted into a standardised Excel form detailing author, year, country, study design, sample size, age at surgery, follow-up duration, mean myopic shift (D), axial length change (mm), predictors analysed, and direction or significance of association of each included study. Extraction accuracy was verified independently by all reviewers (IM, ND, MB, EO).

### 2.6. Data Synthesis

Due to methodological heterogeneity across studies in design, follow-up duration, and outcome reporting, a narrative and tabular synthesis was undertaken. Results were summarised descriptively and organised by age at surgery, follow-up duration, and principal predictors of myopic shift. Quantitative data were presented as mean ± standard deviation where available. No statistical pooling or quantitative meta-analysis was performed. Generative artificial intelligence tools were used solely for language refinement during manuscript preparation and did not contribute to study selection, data extraction, analysis, or interpretation.

## 3. Results

### 3.1. Study Characteristics

A full list of abbreviations related to refractive and biometric parameters is provided in Supplementary Table S1. This review analysed twelve studies investigating postoperative refractive change and its determinants following paediatric cataract surgery with IOL implantation. Studies were conducted across ten countries, with the largest number originating from China [6,11], followed by Brazil [3,13] and Saudi Arabia [2,10] (two studies each). Single studies were identified from India [1], Turkey [5], Latvia [7], Hong Kong [9], Peru [4], and the United States [8]. The earliest study period commenced in 2003 [13], and the most recent data collection extended to 2024 [6,10,11], reflecting two decades of international research activity.

### 3.2. Study Design and Population

Of the twelve included studies (please refer to Table 1), nine were retrospective cohort designs and three were prospective or longitudinal. Sample sizes ranged from 22 to 252 eyes. The mean or median age at surgery ranged from 3.5 months to 10.6 years, with the youngest group represented by the *Long-term outcomes following primary intraocular lens implantation in infants younger than 6 months* (USA) study and the oldest by the *Understanding post-operative refractive outcome in paediatrics after IOL implantation* (Saudi Arabia) cohort [1,2]. Follow-up durations varied between 2 and 15.9 years, with the longest follow-up reported in the Hong Kong study (*Ten-year refractive and visual outcomes of intraocular lens implantation in infants with congenital cataract* [9]).

### 3.3. Reported Refractive and Biometric Outcomes

All twelve studies reported postoperative change in spherical equivalent (SE) as the principal outcome (refer to Table 1). Seven studies additionally assessed axial length (AL) change, and three provided data on keratometry (K) variation or astigmatism (ΔK). Across studies, the reported magnitude of postoperative myopic shift ranged from approximately −1.8 D after 2 years to −11.6 D after 15 years. The greatest shifts were documented in infants operated under 6 months of age, with mean SE change between −5.7 D and −7.5 D within the first 3–5 years postoperatively [1,13]. Axial elongation, where recorded, ranged between +1.7 mm and +5.8 mm over follow-up [4].

### 3.4. Age, Laterality, and Biometric Predictors

Age at cataract extraction or IOL implantation was the most consistently examined parameter, evaluated in all twelve studies. Eleven studies reported a greater magnitude of myopic shift in younger age groups [1,2,3,4,6,7,8,9,10,11,13]. Laterality was analysed in eight studies: five documented larger shifts in unilateral than bilateral cases [1,3,4,5,6]. Axial length was assessed in ten studies, with shorter preoperative AL associated with larger postoperative refractive change in seven [4,6,8,9,10,11,13]. Corneal curvature was evaluated in three studies, with flatter mean K values corresponding to greater myopic shift [2,6,7].

### 3.5. Surgical and Technical Parameters

IOL power, type, implantation site, and calculation formula were reported in varying detail across studies. Three studies compared primary versus secondary IOL implantation, finding similar mean refractive changes over time [5,10,13]. The SRK/T, Holladay 1, and Hoffer Q formulas were the most frequently used; five studies specifically analysed prediction error (PE) or absolute prediction error (APE) as measures of calculation accuracy [2,6,8,11,13]. Mean PE ranged between +0.24 D and +0.70 D, and mean APE between 1.3 D and 1.7 D, with higher variability in younger age groups. Studies using Holladay 1 or IOL Master-based calculations generally reported smaller APE values compared with manual A-scan methods [2,11,13].

### 3.6. Postoperative Complications

Postoperative complications were reported in nine studies. The most frequent were visual axis opacification (13–18%), glaucoma (up to 19%), and strabismus [1,3,5,8,10,13]. Other reported events included posterior capsule rupture, IOL decentration, and synechiae [3,5,10,13]. In studies that provided subgroup data, eyes with postoperative complications exhibited larger mean myopic shifts than uncomplicated cases [1,3,10,13].

### 3.7. Summary of Reported Evidence

**Figure 2:**
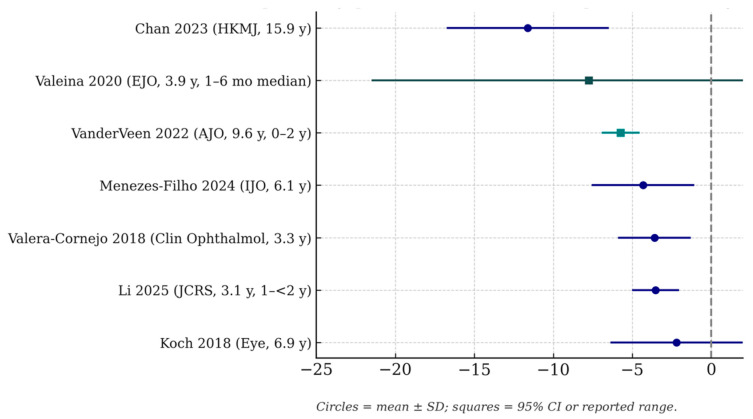
Reported myopic shift in paediatric pseudophakia across major cohorts [3,4,6,7,8,9,13]. Negative values indicate greater myopic shift (D).

All included studies documented measurable postoperative refractive change following paediatric cataract surgery with IOL implantation. The variation in magnitude reflected differences in patient age, laterality, biometric profiles, IOL calculation methods, and duration of follow-up (Figure 2).

The magnitude of postoperative refractive change (mean myopic shift (D)) reported in key studies. Each circle represents the mean ± standard deviation, while squares indicate the 95% confidence interval or reported range where applicable. Follow-up duration (in years) is indicated beside each study. Across cohorts, reported myopic shift ranged from approximately −3 D to −20 D depending on age at surgery, follow-up length, and cohort composition.

## 4. Discussion

Research over the past two decades has demonstrated that postoperative refractive change in children with IOL implantation is not static but evolves throughout ocular growth. The twelve studies reviewed here illustrate how this process, often termed “myopic shift,” is influenced by a complex interplay between age at surgery, ocular biometry, surgical strategy, and postoperative events.

### 4.1. Factors Influencing Refractive Change After Surgery

Across international cohorts, a consistent pattern of refractive drift toward myopia was documented. The extent of this shift ranged widely, from small changes of less than two diopters in later childhood to large shifts exceeding ten diopters in eyes implanted during infancy. Long-term data from Hong Kong, the United States, and India reveal that the steepest refractive change occurs within the first few postoperative years, after which the rate of progression slows but seldom stabilises completely [1,9,13]. This pattern mirrors physiological eye growth, reinforcing that even with lens implantation, paediatric eyes continue to elongate throughout early development [1,4,8,9,11].

1. Age and Axial Growth

Age at implantation emerged as the most reproducible determinant across the literature [2,6,7,11]. Younger infants consistently demonstrated greater myopic progression, confirming that the timing of surgery is pivotal in refractive planning [1,4,6,7,9,11,13]. Where longitudinal biometry was available, axial elongation tracked closely with the degree of refractive change; increases of 2–6 mm was recorded over follow-up periods extending to adolescence [4]. Shorter preoperative axial length was also associated with larger shifts, suggesting that eyes smaller at baseline undergo proportionally greater post-surgical growth [4,6,11].

2. Laterality, Corneal Curvature, and Inter-Eye Factors

Unilateral cataract cases presented additional refractive challenges [6]. Five of eight studies comparing unilateral with bilateral surgery reported larger myopic drift in the operated eye, particularly when inter-ocular axial length differences were small preoperatively [1,4,5,6,13]. These findings have important clinical implications for long-term anisometropia management, highlighting the need for careful refractive targeting, close longitudinal follow-up, and proactive optical correction strategies to support visual rehabilitation in unilateral paediatric pseudophakia. Corneal curvature data, although limited, followed a similar trend—flatter keratometry values were linked to greater refractive change, indicating that anterior segment geometry may also contribute to postoperative outcomes [8,11].

3. Surgical Technique and Biometric Accuracy

Technical choices during surgery and lens power calculation substantially influenced refractive outcomes [6,11]. Comparisons between primary and secondary IOL implantation showed broadly similar average shifts but greater variability in secondary cases due to wider age ranges [5,13]. Optical biometry and modern formulas such as Holladay 1 tended to yield lower absolute prediction errors than manual A-scan or SRK II calculations, while eyes with high astigmatism showed greater residual error [2,11,13]. Across the literature, mean absolute prediction error typically fell between 1.3 D and 1.7 D, demonstrating the persistent difficulty of achieving precise refractive targeting in growing eyes [2,6,11].

### 4.2. Complications and Postoperative Course

Postoperative adverse events were common and variably reported. Visual axis opacification, glaucoma, and strabismus accounted for most complications, and when subgroup data were available, these conditions were linked to larger average myopic shifts [1,8,13]. Given that younger age at surgery is associated both with faster physiological ocular growth and a higher incidence of postoperative complications, age is likely a shared underlying driver for both phenomena, with complications acting as potential modifiers rather than independent causes of refractive change [12]. Although definitions and follow-up intervals differed across studies, the association suggests that inflammatory or structural sequelae may exacerbate ongoing refractive change [1,3,10,13].

### 4.3. Methodological Variability in Study Design and Reporting

Methodological diversity remains the principal limitation of the available evidence. The twelve included studies differed in design, follow-up duration, refraction reporting format, and biometry technique [8]. Only a small proportion were prospective [8,9,13]. Calculation formulas developed for adult eyes were commonly applied to paediatric populations without age-specific adjustment [2,6,11,13]. Such variability precludes meta-analytic synthesis and complicates cross-comparison of refractive outcomes.

### 4.4. Context Within Global Practice

Most published data originate from Asia and South America, reflecting regional differences in timing of cataract detection and surgical thresholds [1,2,3,4,6,9,10,11,13]. Mean age at implantation varied by more than two years between Asian and Western cohorts, and baseline ocular dimensions also differed [1,4,6,8,9,11,13]. These variations demonstrate the need for region-specific reference data on axial and refractive growth following lens implantation.

### 4.5. Future Research and Clinical Implications

Collectively, current studies suggest that postoperative myopic shift is a predictable, age-dependent process rather than a surgical failure [1,2,3,4,5,6,7,8,9,10,11,13]. Standardised biometry refers to the consistent use of optical axial length and keratometry measurements, predefined age-stratified refractive targets, and uniform reporting of myopic shift and prediction error at fixed postoperative intervals [12]. Refining refractive planning according to such standardised parameters therefore depends on accurately modelling ocular growth rather than eliminating refractive change altogether. Future research should prioritise prospective, multi-centre designs with standardised biometry, unified outcome definitions, and age-stratified target refraction strategies. Incorporating modern optical biometry, contemporary IOL power formulas, and longitudinal tracking of axial length will be essential to improve prediction accuracy and to inform consensus guidelines on under-correction in early surgery.

## 5. Conclusions

This scoping review demonstrates that postoperative myopic shift is a consistent and physiologically driven feature of paediatric pseudophakia, with the greatest refractive change occurring following surgery in early infancy. Age at implantation, axial growth dynamics, and biometric measurement accuracy are the most influential factors affecting postoperative refractive outcomes, while unilateral disease and postoperative complications introduce additional variability. Standardised longitudinal biometry, harmonised refractive reporting, and age-stratified refractive targeting strategies are needed to optimise IOL selection and support more predictable long-term refractive outcomes in children undergoing cataract surgery.

## Figures and Tables

**Table 1:** Study characteristics and predictive factors for myopic shift in paediatric pseudophakia.

Study (Author, Year)	Country	Design	Sample (Eyes)	Age at Surgery (Months/Years)	Follow-Up (Years, Months)	Primary Outcome (Myopic Shift, D)	Secondary Outcome (AL change, mm)	Predictive Parameters Tested	Main Findings, Direction of Association
Koch et al., 2018 (Eye) [13]	Brazil, Spain	Retrospective cohort	75	94 ± 45 days; secondary IOL implantation at 20.7 ± 6.0 months (range 11–30)	6.9 years (82.3 ± 48.9 months; range 13–189)	At 30 days +2.16 ± 3.12 D → −2.20 ± 4.19 D at last follow-up; glaucomatous eyes excluded: −1.72 ± 3.66 D. Longer follow-up correlated with greater myopic SE	Axial length at IOL implantation 20.27 ± 1.46 mm; not longitudinally compared but correlated with refractive change	Age at cataract surgery and IOL implantation, formula type (SRK-II vs. SRK/T), IOL power, glaucoma status, laterality	Younger cataract extraction age (<2 mo) and use of SRK-II formula predicted more myopic final SE. Secondary IOL ≤ 20 mo not a glaucoma risk. Secondary IOL under 30 mo gave stable long-term refraction with predictable mild MS
Negalur et al., 2018 (Indian J Ophthalmol) [1]	India	Retrospective, observational cohort	69	4.6 months (range 1.5–6)	4.2 years (range 3–7)	−6.7 D over 4.2 years (≈−2.0 D/year). Median SE: +5.75 D immediate postop → −0.25 D at 3 years → −1.31 D at 5 years (total ≈ −8.4 D). Greater shift in unilateral vs. bilateral	Bilateral +1.73 mm; unilateral +2.80 mm at 3 years	Age, gender, laterality, IOL undercorrection, baseline AL, postoperative complications (VAO, deposits, glaucoma)	Unilateral cataracts showed significantly higher myopic shift and AL elongation. No significant association between age, gender, AL, or IOL undercorrection and final VA. VAO most common complication (18.8%), followed by IOL deposits (15.9%) and glaucoma (2.9%). Supports safety of primary IOL implantation <6 months with predictable large MS
Valera-Cornejo & Flores Boza, 2018 (Clin Ophthalmol) [4]	Peru	Retrospective cohort	76	25.3 ± 16.5 months (range < 48 mo; all < 4 y)	3.3 years (39.7 ± 14.1 months)	3.6 ± 2.3 D; AL > 21.5 mm = 3.2 ± 3.3; AL ≤ 21.5 mm = 3.9 ± 3.2 (*p* = 0.36). Unilateral cataracts = 6.3 ± 6.2 D; bilateral = 3.0 ± 1.9 D (*p* = 0.001). Bilateral subgroup: AL > 21.5 mm = 2.6 ± 2.0 vs. AL ≤ 21.5 mm = 3.4 ± 1.8 (*p* = 0.098). MS increased with follow-up time: 1.95 D (1–2 y), 3.46 D (2–3 y), 3.84 D (3 y)	Mean preoperative AL 21.2 ± 2.4 mm; no significant correlation between AL and shift	Preoperative AL, laterality, age at surgery, visual axis obscuration, follow-up time	Laterality strongly associated with MS: unilateral eyes had >2× greater shift than bilateral. No significant correlation between initial AL and MS overall. Trend toward greater shift with smaller AL in bilateral cataracts. Visual axis obscuration not significant. Concluded that unilateral cases need more hyperopic target refraction
Valeina et al., 2020 (European J Ophthalmol) [7]	Latvia	Retrospective cohort	137	Grouped by age (months) at IOL implantation: 1–6, 7–12, 13–24, 25–48, 49–84; 7–18 years	3.9 years (range 0.5–10)	1–6 mo group median −7.75 D (−21.5 to +2.0); 7–12 mo −3.0 D (−8.75 to +2.0); 13–24 mo −2.5 D (−7.5 to +0.5); ≥25 mo ≈ 0 to −1 D	Not numerically reported; trend of greater AL elongation in younger groups and eyes with SG	Age at surgery, IOL power, morphology, laterality, SG, SC, refraction target, BCVA	Younger age at surgery strongly predicted larger MS. Higher IOL power correlated negatively with shift. Eyes with SG had greater MS (−6 D vs. −2.75 D). No difference between emmetropic and hypermetropic targets. VA correlated with magnitude of MS. Largest shift in diffuse/total and nuclear cataracts; earlier surgery produced 3× greater MS than later groups.
VanderVeen et al., 2022 (Am J Ophthalmol) [8]	USA	Longitudinal cohort	162	Grouped by age: 0–2, 2–4, 4–6, 6–8, 8–10 years	Median 9.6 years (IQR 7.3–12.2)	0–2 y −5.75 D (−6.94, −4.53); 2–4 y −2.25 D (−3.56, −0.94); 4–6 y −1.56 D (−2.81, −0.53); 6–8 y −0.06 D (−0.91, 0.12); 8–10 y 0.00 D (0.00, 0.00)	Not directly quantified per group; RRG: 0–2 y −11, 2–4 y −10, 4–6 y −10, 6–8 y −11, 8–10 y −6	Age, sex, laterality, AL percentile, K, IOL power, follow-up duration	Younger age → greater MS. Lower K (<25th percentile) independently predicted more MS and faster refractive growth. No significant association with AL percentile, laterality, or sex after adjustment. Proposed refined postoperative hyperopia targets by age and K. Greater shift in flatter corneas
Chan et al., 2023 (Hong Kong Med J) [9]	Hong Kong	Retrospective cohort	22	5.3 ± 2.4 months (1.8–10.5)	15.9 ± 2.8 years (10–20.5)	10-year = −11.62 ± 5.14 D (−21.88 to −3.75)	+5.83 ± 2.05 mm increase (17.72 → 24.80 mm)	Age at surgery, immediate post-op refraction, AL, laterality, need for posterior capsulotomy	Younger age → greater MS at 1 and 10 years. Greatest MS during first year. AL change correlated with MS. Immediate post-op refraction ≥ +7 D linked to worse BCVA
Kaplan et al., 2023 (Turk J Med Sci) [5]	Turkey	Retrospective and comparative cohort	242	Primary IOL: 5.9 ± 3.3 years (2–15 y); Secondary IOL: 0.4 ± 0.3 years (0.08–1.5 y; IOL at 2.8 ± 0.4 y)	Primary IOL: 5.9 ± 3.3 years (2–15 y); Secondary IOL: 0.4 ± 0.3 years (0.08–1.5 y; IOL at 2.8 ± 0.4 y) Primary IOL median 5 years (range 4–16); Secondary IOL median 8 years (range 4–19)	No significant difference in MS between groups (*p* = 0.172); Myopia in 57.7% of secondary vs. 48.9% of primary IOL eyes. In unilateral cases, greater myopic shift in operated vs. fellow eyes	AL not measured longitudinally; shift presumed optical rather than axial	Age at surgery, type of IOL (primary vs. secondary), laterality, strabismus, nystagmus, binocular vision, VAO, SG	Primary IOL eyes showed better BCVA and less strabismus/nystagmus, but similar MS compared to secondary IOLs. Unilateral cases in both groups had greater MS than fellow eyes. Early surgery not associated with less nystagmus. Suggests delayed secondary IOL reduces high myopia risk when optical correction compliance is good
Aldamri et al., 2024 (Saudi J Ophthalmol) [10]	Saudi Arabia	Retrospective cohort	202	≤4 years old at surgery (mean age at presentation 15–16 months)	Minimum 3 years (up to 8 years)	MS > −4.00 D observed in 14% of eyes, more common with primary IOL implantation (19 eyes) vs. secondary IOL (10 eyes). One case operated at 5 months had −19.00 D at age 6 years	Not quantified numerically; eyes with primary IOL showed greater elongation and more frequent glaucoma	Age at surgery, IOL type (primary vs. secondary), laterality, cataract morphology, strabismus, nystagmus, glaucoma, VAO	MS ≥ −4 D occurred in 14% of eyes, significantly more frequent in primary IOL cases. Early IOL (<6 mo) associated with larger MS and more SG. Glaucoma was most common complication (19%). Presence of nystagmus/strabismus correlated with poorer VA outcomes
AlObaisi et al., 2024 (Int Ophthalmol) [2]	Saudi Arabia	Retrospective and cross-sectional cohort	47 (29 primary IOL, 18 secondary IOL)	6.52 ± 4.61 years (range 1–15)	Two follow-ups: 2 months and 2 years post-op	Postoperative SE: +1.31 ± 2.65 D; at last follow-up −0.53 ± 2.60 D (net MS ≈ −1.84 D over 2 years)	Not measured directly, but AL correlated negatively with postoperative refraction	Age, AL, ΔK, target refraction, preoperative SE, IOL placement, IOL type (primary vs. secondary), calculation method (Holladay 1 vs. SRK/T)	High ΔK and sulcus IOL placement increased APE. Calculation method strongly influenced outcomes: Holladay 1 yielded lower PE/APE than SRK/T. AL and age correlated negatively with PE and postoperative SE, indicating reduced refractive error in older children. Concluded calculation method and astigmatism are strongest predictors of postoperative refractive error
Li et al., 2024 (TVST) [11]	China	Retrospective cohort	222	4.36 years (IQR 3.16–6.0)	Median 4.18 years (IQR 3.48–4.64)	Myopic shift (D) by age × AL subgroup 2–<4 y: <25% −3.00 D (−4.38, −2.50); 25–75% −2.63 D (−4.63, −1.00); >75% −1.38 D (−2.13, −0.50). 4–<6 y: <25% −3.00 D (−3.63, −1.75); 25–75% −1.50 D (−2.13, −1.13); >75% −0.88 (−2.06, 0.06). ≥6 y: <25% −2.50 D (−5.13, −1.88); 25–75% −1.38 D (−3.19, −1.00); >75% −0.50 D (−0.88, 0.13)	Not specifically quantified, but shorter AL associated with greater elongation and shift	Age at surgery, preoperative AL, K, IOL position, VAO, Nd:YAG, follow-up	Younger age and shorter preoperative AL predicted greater MS and faster rate. No effect of K or IOL position. Suggested AL-based target refraction adjustment (+0.5 to +1 D for short AL)
Menezes-Filho et al., 2024 (Indian J Ophthalmol) [3]	Brazil	Cross-sectional retrospective	81	7.7 months (IQR 3.7–30.5)	6.1 years (72.9 ± 37.1 months)	Overall −4.32 D ± 3.25; by age — <6 mo −5.73 D ± 3.14, 6–24 mo −4.00 D ± 3.15, >24 mo −2.52 D ± 2.57. Greater shift in aphakic eyes (−5.57 D) vs. pseudophakic (−3.44 D). Strabismic eyes −4.99 D vs. non-strabismic −2.52 D. Eyes with surgical complications −5.87 D vs. no complications −3.75 D	Not numerically detailed; shorter AL and microphthalmia correlated with greater MS and complications	Age at surgery, bilaterality, strabismus, aphakia/pseudophakia, BCVA, surgical complications, VAO, anterior vitrectomy, microphthalmia, SG, follow-up duration	Multivariate model: younger age and surgical complications independently predicted greater MS. Strabismus, VAO, and aphakia significant only in univariate models. Concluded that early surgery and intraoperative/postoperative complications strongly increase postoperative MS
Li et al., 2025 (J Cataract Refract Surg) [6]	China	Retrospective, single center cohort	252	3.99 ± 1.98 years (range 1.21–11.89)	3.14 ± 0.25	1–<2 y: −3.53 D ± 1.49; 2–<4 y: −3.08 D ± 1.77; 4–<6 y: −1.75 D ± 1.55; ≥6 y: −1.99 D ± 1.80. Fellow eyes showed minimal shift (−0.34 to −1.67 D). Mean interocular difference significant for groups < 6 y	Not measured longitudinally; mean preoperative AL 22.51 ± 1.70 mm (treated) vs. 22.13 ± 1.04 mm (fellow); shorter interocular AL difference (IALD) linked to greater postoperative change	Age at surgery, preoperative AL, AK, IALD, IAKD, IOL power, VAO, Nd: YAG, laterality	Younger age and smaller IALD independently predicted greater MS. No association with preoperative AL, AK, or IOL power. Recommended age- and IALD-adjusted target refractions: +3 to +4 D for 1–<2 y; +3 D for 2 y; +2 D for 3 y; +1 D for 4 y; 0 to +1 D for 4–<6 y; emmetropic for ≥6 y

AL = axial length; AK = anterior keratometry; APE = absolute prediction error; BCVA = best-corrected visual acuity; D = diopters; ΔK = change in keratometry; IALD = interocular axial length difference; IAKD = interocular anterior keratometry difference; IOL = intraocular lens; IQR = interquartile range; K = keratometry; MS = myopic shift; Nd:YAG = neodymium-doped yttrium aluminium garnet; PE = prediction error; RRG = rate of refractive growth; SE = spherical equivalent; SG = secondary glaucoma; SRK-II/SRK/T = Sanders–Retzlaff–Kraff formula; VA = visual acuity; VAO = visual axis obscuration.

## Data Availability

All data generated or analysed during this study are included in the published article and its Supplementary Materials.

## Supplementary Materials

The following supporting information can be downloaded at: https://www.mdpi.com/article/10.3390/medicina62010106/s1, Table S1: Complete list of abbreviations and definitions used in the scoping review.

## References

[1] Negalur M., Sachdeva V., Neriyanuri S., Ali M.H., Kekunnaya R. (2018). Long-term outcomes following primary intraocular lens implantation in infants younger than 6 months. Indian J. Ophthalmol..

[2] AlObaisi S., Alnasser B.N., Almuhawas H.A., Alhoshan S.A., Aldebasi M.H., Alshaye R., Aldakhil S., Alrasheed S.H. (2024). Understanding post-operative refractive outcome in paediatrics after IOL implementation: Factors and predictors. Int. Ophthalmol..

[3] Menezes Filho C., Messias A., Silva P.H.F., Antunes-Foschini R. (2024). Myopic shift in paediatric cataract surgery associated with age and surgical complications. Indian J. Ophthalmol..

[4] Valera Cornejo D.A., Flores Boza A. (2018). Relationship between preoperative axial length and myopic shift over 3 years after congenital cataract surgery with primary intraocular lens implantation at the National Institute of ophthalmology of Peru, 2007–2011. Clin. Ophthalmol..

[5] Kaplan A.T., Öskan Yalçin S., Oral Aydin A.Y. (2023). Primary versus secondary intraocular lens implantation following removal of congenital/developmental cataracts: Outcomes after at least 4 years. Turk. J. Med. Sci..

[6] Li Y., Jin G., Tan Y., Chen H., Jin J., Luo L., Chen W., Lin H., Liu Y., Liu Z. (2025). Myopic shift after primary intraocular lens implantation in unilateral cataract children and its association with preoperative ocular parameters. J. Cataract. Refract. Surg..

[7] Valeina S., Heede S., Erts R., Sepetiene S., Skaistkalne E., Radecka L., Vanags J., Laganovska G. (2020). Factors influencing myopic shift in children after intraocular lens implantation. Eur. J. Ophthalmol..

[8] VanderVeen D.K., Oke I., Nihalani B.R. (2022). Deviations From Age-Adjusted Normative Biometry Measures in Children Undergoing Cataract Surgery: Implications for Postoperative Target Refraction and IOL Power Selection. Am. J. Ophthalmol..

[9] Chan J.J.T., Wong E.S., Lam C.P.S., Yam J.C. (2023). Ten-year refractive and visual outcomes of intraocular lens implantation in infants with congenital cataract. Hong Kong Med. J..

[10] Aldamri A., AlKhaldi S.A., AlQahtani D.S., AlShaalan K.S., Alshamrani M. (2024). Long-term outcome and determinants of primary paediatric cataract surgery. Saudi J. Ophthalmol..

[11] Li Y., Tan Y., Xu C., Jin G., Chen H., Jin L., Luo L., Chen W., Lin H., Liu Y. (2024). Association Between Preoperative Ocular Parameters and Myopic Shift in Children Undergoing Primary Intraocular Lens Implantation. Transl. Vis. Sci. Technol..

[12] Lloyd I.C., Ashworth J., Biswas S., Abadi R.V. (2007). Advances in the management of congenital and infantile cataract. Eye.

[13] Koch C.R., Kara-Junior N., Serra A., Morales M. (2018). Long-term results of secondary intraocular lens implantation in children under 30 months of age. Eye.

