# Supplementary material for: Predicting Postoperative Myopic Shift After Paediatric Intraocular Lens Implantation: A Scoping Review of Associated Factors
Source: Medicina (Kaunas). 2026 Jan 3;62(1):106. doi: 10.3390/medicina62010106 (PMC12843486; doi:10.3390/medicina62010106)
Supplement: Supplementary file 1 [file medicina-62-00106-s001.zip › medicina-4054262-supplementary.pdf]

Supplementary Table S1. Complete list of abbreviations and definitions used in the scoping review
**AL (axial length)** – The distance from the anterior cornea to the retinal pigment epithelium, which determines the eye's optical power and strongly influences refractive status.
**AK (anterior keratometry)** – A measurement of the curvature of the anterior corneal surface used to estimate corneal refractive power.
**APE (absolute prediction error)** – The absolute difference between predicted and achieved postoperative refraction, used to assess IOL calculation accuracy.
**BCVA (best-corrected visual acuity)** – The sharpest vision a patient can achieve using optimal refractive correction such as glasses or contact lenses.
**D (diopters)** – The unit of measurement of optical power used to quantify refractive error and lens strength.
**ΔK (change in keratometry)** – The postoperative change in corneal curvature, often reflecting surgical or developmental alterations in corneal shape.
**IOL (intraocular lens)** – An artificial lens implanted in the eye to replace the natural crystalline lens, typically after cataract removal.
**IOLMaster** – A non-contact optical biometer that measures axial length, keratometry, and other parameters used for IOL power calculations.
**IQR (interquartile range)** – A statistical spread describing the middle 50% of a dataset, representing the difference between the 75th and 25th percentiles.
**K (keratometry)** – A measurement of corneal curvature that determines the cornea's contribution to the eye's refractive power.
**MS (myopic shift)** – The postoperative increase in myopia over time due to ocular growth or axial elongation in pediatric pseudophakia.
**Nd:YAG (neodymium-doped yttrium aluminium garnet)** – A laser commonly used to perform posterior capsulotomy or treat secondary opacification.
**OSF (Open Science Framework)** – An online platform for preregistration, data sharing, and archiving of research protocols and datasets.
**PE (prediction error)** – The signed difference between predicted and achieved refractive outcome, indicating whether postoperative refraction is more myopic or hyperopic than intended.

**PFV (persistent fetal vasculature)** – A congenital anomaly caused by failure of the fetal hyaloid vasculature to regress, often complicating cataract surgery.
**PRISMA-ScR (Preferred Reporting Items for Systematic Reviews and Meta-Analyses extension for Scoping Reviews)** – A reporting guideline that standardises methodology and transparency in scoping reviews.
**RRG (rate of refractive growth)** – A measure describing the speed at which refractive error changes in growing pediatric eyes over time.
**SC (subcapsular cataract)** – A cataract located just beneath the lens capsule, often affecting visual clarity depending on its density and location.
**SD (standard deviation)** – A statistical measure indicating the amount of variability or dispersion within a set of numerical data.
**SE (spherical equivalent)** – A single value summarising refractive error, calculated as the sphere plus half the cylinder.
**SG (secondary glaucoma)** – A postoperative form of glaucoma developing as a complication of pediatric cataract surgery or IOL implantation.
**SRK-II / SRK/T (Sanders–Retzlaff–Kraff formulas)** – Generations of IOL power calculation formulas that use axial length and keratometry to predict appropriate lens power.
**TVST (Translational Vision Science & Technology)** – A peer-reviewed journal focusing on research bridging basic visual science and clinical application.
**VA (visual acuity)** – A measure of the eye’s ability to resolve detail, typically assessed with standardized charts.
**VAO (visual axis obscuration)** – Any opacity, such as posterior capsule opacification, that blocks the visual axis and degrades retinal image quality.
